# Appropriate Waist Circumference Cutoff Values for Persons with Multiple Cardiovascular Risk Factors in Japan: a Large Cross-sectional Study

**DOI:** 10.2188/jea.18.37

**Published:** 2008-02-28

**Authors:** Sachiko Narisawa, Kazutoshi Nakamura, Kiminori Kato, Kazumi Yamada, Juei Sasaki, Masaharu Yamamoto

**Affiliations:** 1 Department of Nursing, School of Health Sciences, Niigata University; 2 Department of Community Preventive Medicine, Niigata University Graduate School of Medical and Dental Sciences; 3 Division of Cardiology, Niigata University Graduate School of Medical and Dental Sciences; 4 Plaka Health Care Center, Niigata Medical Association for Labor Health

**Keywords:** Cross-Sectional Studies, Japan, Metabolic Syndrome X, Risk Factors, ROC Curve

## Abstract

**Background:**

In Japan, the current standard waist circumference cutoff value for persons with multiple cardiovascular risk factors remains controversial. In this study we aimed to analyze the health-check examination data from a large Japanese population and propose a revised waist circumference cutoff value.

**Methods:**

Subjects of this study were 12,725 adults who underwent a health-check by thorough medical examination between April 2006 and March 2007. Medical examinations included measurement of waist circumference, fasting blood triglycerides, HDL cholesterol, glucose concentrations, blood pressure and collection of demographic characteristics. Receiver operating characteristic (ROC) curve analysis was utilized to find appropriate waist circumference cutoff values in relation to multiple cardiovascular risk factors with two or more of the following: dyslipidemia (hypertriglyceridemia or low HDL cholesterol), hypertension, and hyperglycemia defined by the Japanese criteria of metabolic syndrome.

**Results:**

The average age of the subjects was 50.7 years (standard deviation [SD]: 8.8) for men and 49.7 years (SD: 8.6) for women. ROC curve analysis showed maximum sensitivity plus specificity at a waist circumference of 87 cm in men (0.66 and 0.62, respectively) and 83 cm in women (0.73 and 0.70). When analyzed by ten-year age groups, the ROC curves for younger age groups were shifted up and to the left compared to older age groups, but associations between cutoff values and age were not clear.

**Conclusion:**

In Japan, the appropriate cutoff value of waist circumference for persons with multiple cardiovascular risk factors is 87cm for men and 83 cm for women.

## INTRODUCTION

Metabolic syndrome (MetS) is characterized by multiple risk factors, including abnormal fat distribution, dyslipidemia, hypertension, and hyperglycemia, in relation to insulin resistance and obesity, and persons with MetS are considered to have high risk of cardiovascular diseases (CVD)^1^^,^^2^ Because of the increase of CVD worldwide, the importance of MetS has been enhanced for the prevention and management of CVD, and diagnosis of MetS is expected to make a clinical intervention for the prevention of CVD in high-risk populations.

The diagnostic criteria of MetS has been considered from late 1990s and established lately by several groups, such as the World Health Organization, European Group for the Study of Insulin Resistance (EGIR), and National Cholesterol Education Program-Adults Treatment Panel (NCEP-ATPIII).^1^ These diagnostic criteria have some differences in concept and items, and application of these diagnostic criteria should be cautious to other ethnic groups than whites in European and North American countries. In response to this need, the eight Japanese academic societies in clinical medicine developed a set of diagnostic criteria for MetS in Japanese people.^3^ The Japanese criteria recommend an umbilical level waist circumference cutoff of 85 cm for men and 90 cm for women, but this definition has proved controversial.^4^^-^^6^

Waist circumference is a proxy for visceral obesity, an important component of MetS.^1^ Receiver operating characteristic (ROC) curve analysis has been a useful tool in the study of waist circumference cutoffs,^6^^-^^8^ and has been used in two recent studies^6^^,^^7^ of Japanese populations. These study populations, however, were relatively small (n=692 and n=749), and the researchers did not measure waist circumference at the umbilical level as recommended by the Japanese criteria. We utilized ROC curves to analyze umbilical level waist circumference cutoffs in a large Japanese population (n=12,725). The purpose of this study was to propose waist circumference cutoff values for persons with multiple cardiovascular risk factors in Japanese adults.

## METHODS

### Subjects

Subjects of this study were 12,725 adults who voluntarily came to Niigata Medical Association for Labor Health, Plaka Health Care Center (Niigata, Japan) and underwent a health check-up with thorough medical examination (*Ningen Dock*) between April 2006 and March 2007. All of the subjects were residents of Niigata City or the surrounding areas. We analyzed an anonymous data set including demographic and medical data for all subjects. The protocol of this study was approved by the Ethics Committee of Niigata University School of Medicine.

### Blood Examination

Twelve-hour fasting blood in the morning was drawn from all of the subjects. Serum was obtained, and biochemical measurements were conducted in a routine manner in the laboratory of Niigata Medical Association for Labor Health. The serum triglycerides (Enzymatic method) and HDL-cholesterol (Direct method) concentrations were measured using an auto-analyzer 7700 (Hitachi Co. Ltd, Tokyo, Japan), and the plasma glucose (Hexokinase method) concentration was measured with BioMajesty JCA-BN9030 (JEOL Ltd, Tokyo, Japan).

### Other Measurements

Age, sex, weight, and height were recorded. Body mass index was calculated by weight (kg) divided by the square of height (m^2^). Abdominal obesity was evaluated with measuring abdominal circumference at the level of the umbilicus^3^ by trained nurses. Systolic and diastolic blood pressures were measured using a sphygmomanometer (USM-700GSi of UEDA, Tokyo, Japan), with a subject sitting on a chair after at least a five-minute rest.

### Definition of Multiple Cardiovascular Risk Factors to Obtain Waist Circumference Cutoffs

It is well-accepted that insulin resistance is the essential cause of MetS, and that multiple cardiovascular risk factors of endogenous origin aggregate in one individual in relation to MetS.^9^ Therefore, we considered the presence of multiple cardiovascular risk factors as an outcome variable of the ROC analysis. In this study, an individual was considered to have multiple cardiovascular risk factors if two or more of the following risk factors of metabolic disorders (based on Japanese criteria of MetS) applied: dyslipidemia (triglycerides ≥150mg/dl, HDL-cholesterol <40mg/dl, or current antidyslipidemia medication), hypertension (systolic blood pressure ≥130mmHg, diastolic blood pressure ≥85mmHg, or current antihypertensive medication), and hyperglycemia (fasting plasma glucose ≥ 110 mg/dl or current hypoglycemic medication).^9^ This definition is also used in studies similar to ours that have been published recently.^6^^,^^8^

### Statistical Procedures

SAS^®^ statistical software (release 9.1.3) was used for computation. ROC curve analysis was used to find appropriate waist circumference cutoff values in relation to multiple cardiovascular risk factors. We defined the best cutoff value as the value with the highest accuracy that maximizes the Youden's index (sensitivity + specificity - 1).^10^ Areas under ROC curves (AUCs) were computed with the "PLOC LOGISTIC" of the SAS program. Statistical tests for a comparison of AUCs were conducted by the t-test. Values of p<0.05 were considered statistically significant.

## RESULTS

The average age of the subjects was 50.7 years (standard deviation [SD]: 8.8; range: 21-88) for men and 49.7 years (SD: 8.6; range: 21-84) for women. Anthropometric profiles and results of medical examinations of the subjects by sex and 10-year age groups are shown in Table 1.

**Table 1.** Means (standard deviations ) of anthropometric profiles and results of medical examinations by sex and 10 year age groups.

	Age (year)											

	20-29		30-39		40-49		50-59		60-69		70+	
	Men											
	n=25		n=835		n=2642		n=3176		n=955		n=129	
Height (cm)	172.7	(6.0)	173.0	(5.7)	172.1	(5.6)	169.2	(5.7)	166.2	(5.5)	163.0	(6.0)
Weight (kg)	64.6	(10.4)	69.9	(11.0)	69.9	(10.3)	67.4	(9.4)	64.4	(8.1)	60.8	(8.1)
BMI (kg/m^2^)	21.7	(3.4)	23.3	(3.3)	23.6	(3.2)	23.5	(2.8)	23.3	(2.79)	22.9	(2.7)
Waist circumference (cm)	79.0	(8.8)	83.8	(8.5)	85.8	(8.3)	86.3	(7.6)	86.1	(7.3)	85.4	(7.5)
Triglycerides (mg/dl)	80.8	(33.1)	129.7	(92.4)	150.2	(108.9)	135.9	(92.2)	124.3	(67.2)	101.4	(45.0)
HDL-cholesterol (mg/dl)	57.7	(11.8)	54.7	(13.2)	55.3	(13.6)	56.1	(14.6)	56.2	(14.3)	55.9	(13.2)
Plasma glucose (mg/dl)	85.8	(4.2)	91.4	(14.7)	95.1	(19.0)	99.8	(20.9)	101.8	(23.7)	100.5	(17.6)
Systolic BP (mmHg)	118.3	(9.5)	118.6	(13.1)	122.0	(15.7)	123.8	(16.8)	125.2	(17.2)	126.8	(17.7)
Diastolic BP (mmHg)	73.1	(8.3)	74.2	810.3)	78.9	(11.2)	80.0	(10.5)	78.8	(9.8)	75.1	(10.5)

	Women											
	n=18		n=628		n=1698		n=2131		n=416		n=72	
Height (cm)	158.2	(5.1)	159.8	(5.2)	158.7	(5.2)	155.8	(5.2)	152.6	(5.1)	150.0	(5.5)
Weight (kg)	48.3	(7.3)	53.0	(8.8)	54.6	(9.0)	53.4	(7.8)	52.6	(7.2)	49.7	(7.7)
BMI (kg/m^2^)	19.3	(2.3)	20.8	(3.3)	21.7	(3.4)	22.0	(3.1)	22.6	(3.0)	22.1	(3.1)
Waist circumference (cm)	71.2	(8.6)	76.1	(8.4)	78.8	(9.0)	81.0	(8.5)	83.6	(8.2)	83.1	(8.0)
Triglycerides (mg/dl)	60.2	(17.1)	70.4	(45.5)	75.9	(38.4)	89.6	(48.1)	97.0	(51.8)	97.7	(41.7)
HDL-cholesterol (mg/dl)	68.7	(11.1)	66.5	(13.6)	67.9	(14.8)	67.6	(15.2)	63.6	(14.9)	63.5	(13.2)
Plasma glucose (mg/dl)	83.6	(4.7)	85.7	(7.6)	88.1	(10.4)	91.2	(13.1)	93.6	(15.9)	95.5	(10.2)
Systolic BP (mmHg)	104.6	(9.8)	109.2	(12.1)	114.0	(14.4)	116.6	(15.9)	120.2	(15.8)	125.1	(18.2)
Diastolic BP (mmHg)	64.3	(10.3)	68.4	(8.6)	71.3	(10.1)	73.4	(10.3)	73.3	(10.0)	72.4	(10.3)

BMI: body mass index

HDL-cholesterol: high-density lipoprotein cholesterol

BP: blood pressure

The prevalence of MetS diagnosed by AHA/NHLBI criteria was 1334/7761 (17.2%) for men, 438/4963 (8.8%) for women, and 1772/12724 (13.9%) in total. When using the Japanese criteria, the prevalence was 1180/7762 (15.2%) for men, 105/4963 (2.1%) for women, and 1285/12725 (10.1%) in total.

**Figure 1.**
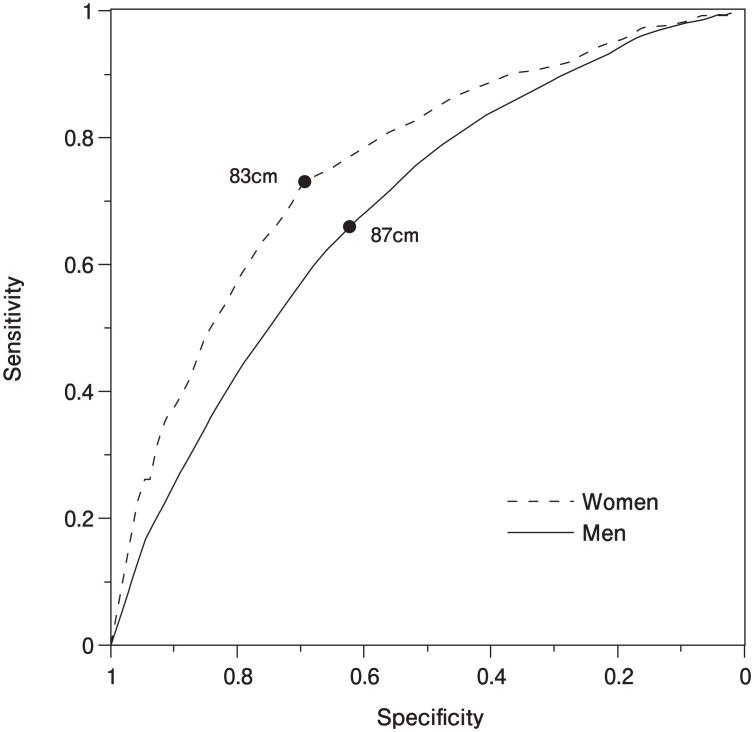
ROC curves of waist circumference by sex in relation to multiple cardiovascular risk factors. Maximum sensitivity plus specificity was achieved at waist circumference cutoffs of 87 cm for men (0.66 and 0.62, respectively) and 83 cm for women (0.73and 0.70). Areas under curves were 0.689 for men and 0.759 for women with a statistically significant difference.

ROC curve analysis by sex suggested that waist circumference is a more sensitive and specific measure of metabolic disease in women than in men (Figure 1). The ROC curve for women was shifted up and to the left compared to the ROC curve for men. These ROC curves achieved a maximum Youden's index at a waist circumference cutoff of 87 cm in men (sensitivity, 0.66; specificity, 0.62) and 83 cm in women (sensitivity: 0.73; specificity: 0.70). Areas under curves were 0.689 for men and 0.759 for women with a statistically significant difference (p<0.001).

**Figure 2.**
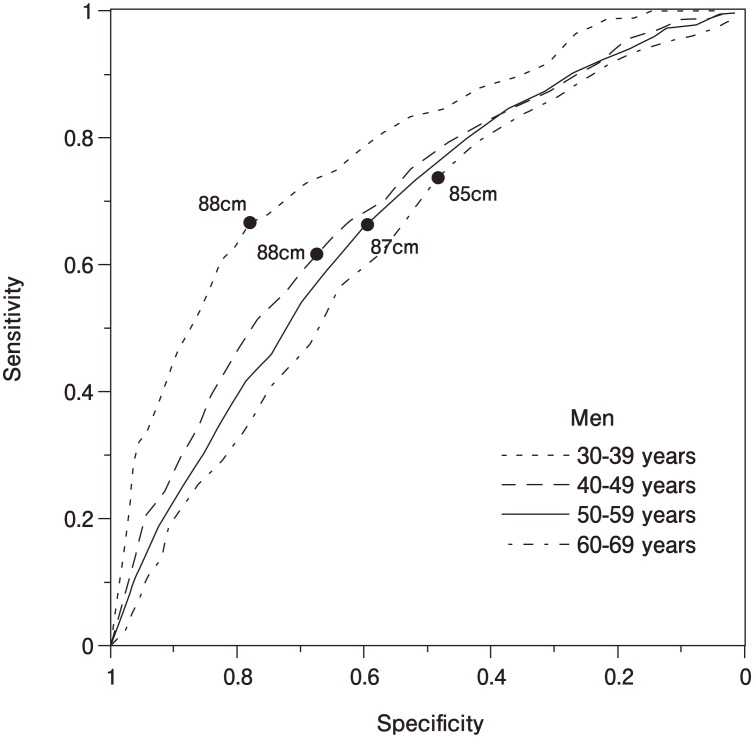
ROC curves of waist circumference in men by ten-year age groups in relation to multiple cardiovascular risk factors. The ROC curves displayed maximum sensitivity plus specificity at waist circumference cutoffs of 88 cm at ages 30-39 (0.67 and 0.78, respectively), 88 cm at ages 40-49 (0.62 and 0.68), 87 cm at ages 50-59 (0.66 and 0.60), and 85 cm at ages 60-69 (0.74 and 0.49). Areas under curves were 0.782 at ages 30-39, 0.696 at ages 40-49, 0.672 at ages 50-59, and 0.635 at ages 60-69 with all pairs being significantly different except for ages 40-49 vs. 50-59 (*p*=0.154) and 50-59 vs. 60-69 (*p*=0.111).

**Figure 3.**
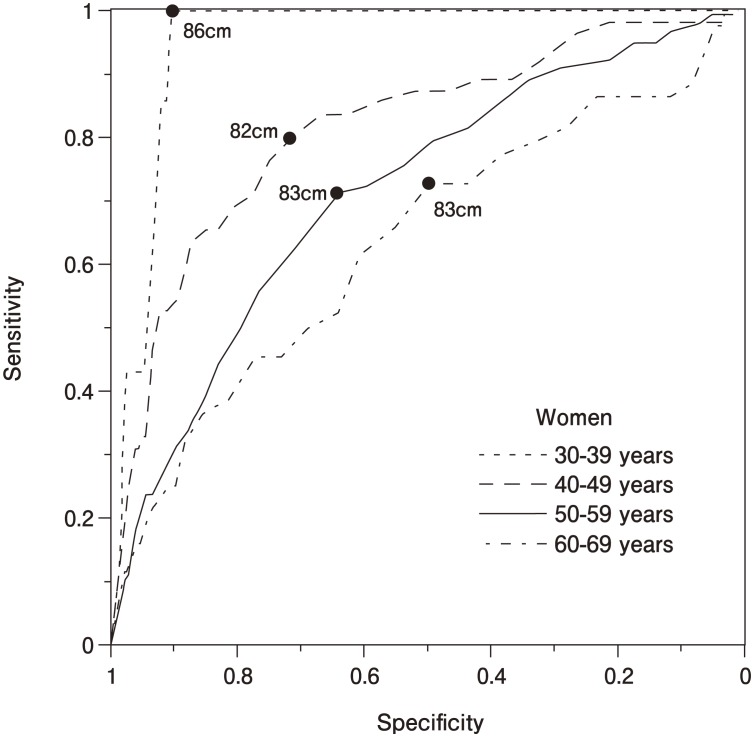
ROC curves of waist circumference in women by ten-year age groups in relation to multiple cardiovascular risk factors. The ROC curves displayed maximum sensitivity plus specificity at waist circumference cutoffs of 86 cm at ages 30-39 (1.00 and 0.91, respectively), 82 cm at ages 40-49 (0.80 and 0.72), 83 cm at ages 50-59 (0.71 and 0.64), and 83 cm at ages 60-69 (0.73 and 0.50). Areas under curves were 0.951 at ages 30-39, 0.816 at ages 40-49, 0.716 at ages 50-59, and 0.636 at ages 60-69 with all pairs being significantly different except for ages 50-59 v.s. 60-69 (*p*=0.138).

Stratified by ten-year age groups, waist circumference ROC curves for younger age groups were shifted up and to the left compared to older age groups in both men (Figure 2) and women (Figure 3), suggesting greater sensitivity plus specificity at younger ages in both sexes. The men's ROC curves (Figure 2) displayed a maximum Youden's index at a waist circumference cutoff of 88 cm at ages 30-39 (sensitivity: 0.67; specificity: 0.78), 88 cm at ages 40-49 (sensitivity: 0.62; specificity: 0.68), 87 cm at ages 50-59 (sensitivity: 0.66; specificity: 0.60), and 85 cm at age 60-69 (sensitivity: 0.74; specificity: 0.49). Areas under curves were 0.782 at ages 30-39, 0.696 at ages 40-49, 0.672 at ages 50-59, and 0.635 at ages 60-69 with all pairs being significantly different (p=0.002 for ages 30-39 vs. 40-49, p<0.001 for ages 30-39 vs. 50-59 and 30-39 vs. 60-69, and p=0.014 for ages 40-49 vs. 60-69), except for ages 40-49 vs. 50-59 (p=0.154) and ages 50-59 vs. 60-69 (p=0.111). The women's ROC curves (Figure 3) showed a maximum Youden's index at a waist circumference cutoff of 86 cm at ages 30-39 (sensitivity: 1.00; specificity: 0.91), 82 cm at ages 40-49 (sensitivity: 0.80; specificity: 0.72), 83 cm at ages 50-59 (sensitivity: 0.71; specificity: 0.64), and 83 cm at ages 60-69 (sensitivity: 0.73; specificity: 0.50). Areas under curves were 0.951 at ages 30-39, 0.816 at ages 40-49, 0.716 at ages 50-59, and 0.636 at ages 60-69 with all pairs being significantly different (p=0.011 for ages 30-39 vs. 40-49, p<0.001 for ages 30-39 vs. 50-59 and 30-39 vs. 60-69, p=0.008 for ages 40-49 vs. 50-59, and p=0.009 for ages 40-49 vs. 60-69), except for ages 50-59 vs. 60-69 (p=0.138).

## DISCUSSION

The major characteristic of rationale behind the Japanese criteria of MetS is that its cutoff values of waist circumference for abdominal obesity (85cm for men and 90cm for women) are different from those of AHA/NHLBI criteria for Asians (90cm for men and 80cm for women). This characteristic of the Japanese criteria is derived from a finding of a firm relationship between insulin resistance and abdominal obesity.^3^ Cutoff values of waist circumference for MetS for Japanese is still controversial,^1^^,^^4^^-^^6^ and thus we conducted ROC analysis to understand waist circumference cutoffs in relation to multiple cardiovascular risk factors of Japanese adults.

Our study showed that cutoff values of waist circumference at the umbilical level of 87 cm for men and 83 cm for women yielded maximum sensitivity plus specificity. Hara et al^6^ and Shiwaku et al^7^ conducted similar studies in relatively small Japanese populations. Hara et al measured waist circumference at mid-abdomen and proposed a cutoff of 85 cm for men and 78 cm for women. Shiwaku et al measured waist circumference at the narrowest level and proposed cutoffs of 82 cm for men and 73 cm for women. Though measurement differences preclude direct comparison, ROC curve analyses in all three studies suggest that the waist circumference cutoff value is lower for women than for men.

Overall, we observed that waist circumference was less sensitive and specific for multiple cardiovascular risk factors in men compared to women. Specificity was as low as 0.66 for men at a waist circumference cutoff of 87 cm (Figure 1). Adoption of a higher waist circumference would improve specificity. For example, to increase waist circumference cutoff specificity to 0.70 (the specificity for women), the men's waist circumference cutoff should be increased from 87 cm to 89 cm.

The large sample size of this study enabled us to stratify our ROC curve analysis by age group. Age group analysis of the ROC curves in this study showed that waist circumference is more sensitive and specific in younger age groups than in older age groups in both sexes. Also, cutoff values for younger age groups, especially for the 30-39-age-group in women, tended to be higher than for older age groups in men and women. At this time, the evidence may support the use of different waist circumference cutoff values for different age groups in men, and different waist circumference cutoff for the 30-39-age-group in women.

There have been a few prevalence studies using the Japanese criteria. Urashima et al^11^ reported that the prevalence of MetS diagnosed by the Japanese criteria in a Japanese population (age-standardized to 2001 National Census Data of Japan) was 14.0% for men and 2.9% for women. By standardizing our data in the same manner, the prevalence of MetS were calculated to be 11.5% for men and 2.2% for women (data not shown in Result). Arai et al^12^ reported the prevalences of 12.1% for men and 1.8% for women (using the Japanese criteria) in a general Japanese population (mean age, 46 years). These previously reported prevalence rates in Japanese adults are similar values, and our results are in agreement with them.

The subjects in this study were not randomly selected from the general population, so we checked whether our sample was biased by comparing the prevalence of obesity (body mass index ≥25 kg/m^2^) and abdominal obesity (waist circumference ≥85 cm for men and ≥90 cm for women) between the subjects in the present study and the ones in the National Health and Nutrition Survey (NHNS) of Japan (Table 2).^13^ We found no evidence of a difference in anthropometric profiles for men, but female subjects (40-69 years of age) in the present study were less obese than their counterparts in the NHNS. Female subjects in this study may have been biased toward lean, and if that were the case, generalization of the results for women in this study should be made with caution; a larger cutoff value of waist circumference for women might be appropriate.

**Table 2.** Prevalence of obesity and abdominal obesity between the subjects in the present study and the National Health and Nutrition Survey (NHNS) in Japan (2004).

	Age (year)			

	30-39	40-49	50-59	60-69
	Men			
Body mass index ≥ 25 kg/m^2^				
This study	27.3	29.1	28.0	26.0
NHNS	28.9	32.7	30.8	29.7

Waist circumference ≥ 85 cm				
This study	39.5	52.4	56.0	56.9
NHNS	37.4	53.2	55.1	59.7

	Women			
Body mass index ≥ 25 kg/m^2^				
This study	9.6	14.2*	14.6*	20.0*
NHNS	8.3	17.9	24.1	29.9

Waist circumference ≥ 90 cm				
This study	6.9	10.3*	13.4*	20.7*
NHNS	5.1	15.1	17.7	27.1

*Significantly different (*p*<0.05) from the NHNS value

The major limitation of this study was that the subjects were a health check-up population, and selection biases may occur in such a setting. Health check-up participants generally more concern about their health than non-participants, and persons with poor health, who consult a family doctor, tend not to participate in health check-up programs. The prevalence of MetS may be underestimated in these circumstances. In fact, female subjects in the present study were shown to be less obese than in the NHNS group. Second, this study used a cross-sectional design, which yields only prevalence. Unlike incidence, prevalence provides limited information on the susceptibility to disease. A longitudinal study should be conducted to determine the relationship between waist circumference and incidence of multiple cardiovascular risk factors.

In summary, we used ROC curve analyses to determine appropriate waist circumference cutoff values for persons with multiple cardiovascular risk factors in Japan. Finding showed that appropriate waist circumference cutoff values are 87 cm for men and 83 cm for women. Since this study was cross-sectional in design, further investigation is needed to confirm whether these cutoffs can be used for MetS diagnosis in Japanese adults.
